# Women’s Satisfaction With and Perceptions of the Quality of Postabortion Care at Public-Sector Facilities in Mainland Tanzania and in Zanzibar

**DOI:** 10.9745/GHSP-D-19-00026

**Published:** 2019-08-22

**Authors:** Colin Baynes, Erick Yegon, Grace Lusiola, Rehema Kahando, Esther Ngadaya, Justin Kahwa

**Affiliations:** a EngenderHealth, Washington, DC, USA.; b EngenderHealth, Nairobi, Kenya.; c EngenderHealth, Dar es Salaam, Tanzania.; d The National Institutes of Medical Research, Dar es Salaam, Tanzania.

## Abstract

**Background:**

In 2015, the government of Tanzania began to strengthen the quality of postabortion care (PAC). Limited research has been conducted to understand clients’ perceptions of public sector provision of PAC. Accordingly, we carried out a mixed-method study between April and July 2016, using client surveys and in-depth interviews, both implemented immediately following PAC. Results were used to help guide the government’s initiative.

**Methodology:**

We assessed the quality of PAC in 25 public-sector facilities through a client survey of 412 women. Questions included satisfaction with client-staff interaction, counseling, provider competence, postabortion family planning, accessibility of care, and the facility environment. Based on responses, we developed and validated a scale representing women’s overall satisfaction with the quality of care. We conducted bivariate analysis to identify the levels of care associated with clients’ ranking of individual and composite measures of the quality of care. We used multivariate ordinal logistic models to assess the relative influence of multilevel factors on clients’ overall satisfaction. We coupled our survey with qualitative analysis of in-depth interviews with 30 PAC clients.

**Results:**

Clients reported moderately high levels of satisfaction with the quality of PAC, with an overall mean score of 2.6 on a 4-point scale. Bivariate analysis identified several areas for improvement, including family planning counseling and provision, especially at regional hospitals; pain management; and reduced use of sharp curettage. The factors most strongly associated with satisfaction were advanced parity, receiving care at lower-level facilities, brief waiting periods, and manual vacuum aspiration for treatment of incomplete abortion. Qualitative analysis illuminated how client-provider interactions; pain; desire for counseling and information, especially on family planning; and congested facility environments shape clients’ perceptions of the care they received.

**Conclusions:**

Although clear areas for improvement in public-sector provision of PAC existed at all sites, women were less likely to report satisfaction with care at referral facilities owing primarily to inadequate counseling, delays in receiving PAC treatment after admission, and poor emphasis on postabortion fertility, family planning information, and contraceptive provision. PAC programs should ensure availability of a wide range of contraceptive methods and high-quality family planning counseling, especially at tertiary facilities.

## Abstract (precis)

Tanzanian women expressed greater satisfaction with postabortion care received at district hospitals and health centers, where they experienced shorter waiting times, more family planning counseling, and threefold greater voluntary uptake of family planning, than at regional hospitals. Continued decentralization to district hospitals would likely enhance client satisfaction with postabortion care.

## INTRODUCTION

Induced abortion occurs frequently in Tanzania, despite legal restrictions against it, and women often undergo the procedure secretly and under unsafe conditions.^1^ In 2013, just over 405,000 induced and spontaneous abortions occurred in Tanzania, yielding a national ratio of 21 total abortions per 100 live births.^2^ Complications include incomplete abortion, hemorrhage, infection, uterine perforation, and damage to the genital tract and internal organs. One study found that for each woman in Tanzania that received postabortion care (PAC) for complications, another 6 did not seek or receive care after an abortion; however, not all of these women experienced complications.^2^ Another study reported that unsafe abortion accounted for 38% of hospitalizations for obstetric complications.^3^ According to 2 other hospital studies, unsafe abortion caused approximately one-quarter of all maternal deaths.^4^^,^^5^

PAC addresses mortality and morbidity from abortion-related complications. As an integrated clinical service package, PAC includes treatment for abortion complications, provision of family planning counseling, and access to a voluntary contraceptive method for healthy spacing of desired pregnancies and avoiding future unintended pregnancies.^6^ PAC includes what has been indicated, screening and treatment of STIs and HIV as well as community empowerment.

The Ministry of Health, Community Development, Gender, Elderly and Children (MOHCDGEC) of Tanzania is strongly committed to PAC, which it includes in its National Package of Essential Health Interventions.^7^ Over time, international donors and NGOs have supported the government in expanding access to this service. A cornerstone of these efforts has been decentralizing PAC from tertiary to lower-level facilities and strengthening of the family planning counseling and service provision component of PAC, emphasizing access to a wider range of methods, especially long-acting reversible contraceptives (LARCs).^8^^,^^9^ To evaluate this process, the World Health Organization recommends assessing clients’ perspectives as part of routine monitoring and evaluation of postabortion services, including postabortion family planning.^10^^–^^12^ Multiple studies indicate that clients’ age is an important factor in PAC delivery. Young women are often more likely to seek an unsafe abortion, develop complications, and either delay or refrain from seeking treatment,^13^ partly because they perceive bias, opprobrium, and discrimination from providers.^14^ A qualitative assessment of women’s views on medical treatment for abortion complications in Zimbabwe found that women preferred misoprostol over vacuum aspiration techniques for PAC, perceiving it as less invasive, safer, and more affordable than surgery.^15^

In our study, we sought to learn about PAC clients’ perception of the quality of care in 25 public-sector facilities in the Mwanza and Geita regions of mainland Tanzania and in Zanzibar, an island archipelago off the coast of the country. Data collection was done within a parent study to obtain baseline information on interventions aimed at improving family planning integration with PAC and accelerating decentralization of the service to lower-level facilities. Past research on the quality of PAC in Tanzania emphasized the feasibility of task shifting to midlevel providers,^16^ the acceptability of postabortion contraception services,^17^ and influences on follow-up care seeking.^18^ Using data from exit interviews and in-depth interviews with PAC clients following their discharge from care, we assessed their satisfaction with the care they received. Our specific objectives were to (1) compare the perceptions of quality among women who received PAC at referral hospitals and lower-level facilities, (2) identify the main drivers of client satisfaction with PAC, and (3) identify protocol elements and client needs that the PAC programs do and do not adequately address.

We sought to learn about PAC clients’ perception of the quality of care in 25 public-sector facilities in mainland Tanzania and Zanzibar.

## METHODS

### Health Systems Context

The MOHCDGEC has authorized the provision of surgical PAC in Tanzania, through both sharp curettage and manual vacuum aspiration (MVA). It has not approved the use of misoprostol, a uterotonic drug, for treatment of incomplete abortion. Zanzibar, which maintains a semi-autonomous relationship with the mainland and has its own Ministry of Health and Social Welfare, permits all these methods for PAC. All supplies and medications for PAC are procured by the MOHCDGEC without U.S. government support. According to global recommendations for PAC treatment of incomplete abortion, sharp curettage should be phased out and replace with MVA and medical methodologies.^10^^,^^11^

### Sampling

We conducted quantitative exit interviews, employing structured questionnaires, with clients from 2 regional referral hospitals, 6 district hospitals, and 9 health centers, all in the public sector, in the Mwanza and Geita regions, and 1 regional referral hospital, 3 district hospitals, and 4 health centers in Zanzibar regions. All regional referral hospitals are in urban areas, and 2 of the 6 district hospitals are close to urban areas near regional capitals in the Mwanza and Geita regions. The remaining facilities are in rural settings. We selected these sites based on data from 2015–2016, which indicated service volume for public-sector facilities. Sites with a PAC client volume of 4 per month were considered eligible for inclusion in this study.

The number of clients recruited at each facility was determined based on the sample size needed to evaluate the effect of an intervention on voluntary postabortion contraceptive uptake before and after the quality improvement intervention. Given the difference in baseline measures for PAC clients’ uptake of a modern contraception method, we made separate calculations for the mainland and for Zanzibar. We took into account the need to measure at 80% power a threefold increase in the proportion of PAC clients that chose a LARC before discharge. We also employed a design effect set at 2 to address clustering and assumed a client refusal or nonresponse rate of 10%. Using population proportionate to size sampling techniques, we established recruitment targets for the proportion of PAC clients to enroll at each facility to reflect service utilization trends during the past year. We subsequently enrolled 228 PAC clients at 17 sites in Mwanza and Geita and 184 at 8 sites in Zanzibar (254 from regional referral sites and 158 from lower-level district hospitals and health centers), for a total of 412 participants.

For the present analysis, we assessed differences in PAC clients’ overall satisfaction with PAC treatment. Informed by stakeholders in the study area, we hypothesized that women receiving PAC at lower-level facilities (district hospitals and health centers) would rate the services more highly than those at referral-level facilities because the latter receive more complex cases and are more crowded. We did not differentiate our sample between the mainland and Zanzibar study populations. Given our sample sizes, we determined that we could detect a 15 percentage-point difference in the proportion of women who classified their level of satisfaction with PAC as high or very high (see the dependent variable below). In both sampling exercises, our a priori significance level was α=.05.

A subset of PAC clients who completed exit interviews were asked to participate in qualitative in-depth interviews (IDIs). For the IDIs, we equally enrolled clients based on their age (18–29 years and 30 years and above) and facility type (regional hospitals and lower-level facilities). Ultimately, 30 PAC clients participated in these interviews.

### Study Instruments and Measures

The exit interview took 45 minutes on average and included binary or categorical questions on sociodemographic characteristics; reproductive health and family formation experiences; recognition of complications; care seeking and social aspects of negotiating access to care; perceptions of the facility environment; features of the client-provider interaction; post-procedure knowledge and recall of counseling; postabortion contraceptive use; and satisfaction with PAC received. The final section of the questionnaire included 12 questions that used Likert scales for ranking the level of satisfaction or agreement with various dimensions of PAC quality, such as waiting time, privacy, cleanliness, and treatment from staff and the PAC providers. Scoring was 1–4 (1, lowest; 4, highest) for each of the questions.

To identify the main influences on client satisfaction with PAC, we created a summary measure of overall satisfaction by combining participants’ scores for each of the 12 domains of satisfaction, respectively. We then developed the dependent variable for our analysis, a 4-point scale representing clients’ overall evaluation of PAC quality. A Cronbach’s alpha coefficient was calculated to validate the scale (α=.80). Based on a recent systematic review of instruments to measure patient experience of health care quality and Quality Criteria for Measurement Properties, our scale was internally consistent and appropriate for our analysis.^19^^,^^20^

To identify the main influences on client satisfaction, we created a summary measure of overall satisfaction.

The IDIs followed an open-ended guide that emphasized the different stages of PAC clients’ experience. The interview was designed to last 30 minutes. Questions pertained to clients’ perceptions of the accessibility of PAC; delays experienced after admission; respectfulness of staff and interpersonal relation with the PAC provider; providers’ technical competence in PAC; the condition of the facility and its environment; choice and availability of services; and satisfaction with counseling and the information received. Data collectors digitally recorded the IDIs and transcribed them in their original Swahili. The transcripts were professionally translated into English, and the study team then entered them into QSR Nvivo-Pro for analysis.

### Study Enrollment

At all sites, PAC providers oriented to the project screened clients who sought treatment for postabortion complications for eligibility to participate based on whether they seemed physiologically and emotionally capable of understanding informed consent procedures. Prior to deployment, 20 data collectors with clinical and social science backgrounds, including experience conducting quantitative and qualitative interviews with women on related topics, received a 10-day training on the ethical, technical, and logistical aspects of data collection. This training included pretesting and refining the survey instrument and IDI guide. The data collectors were deployed from April to June 2016 to recruit PAC clients who had been identified as eligible to participate.

Informed consent included an explanation of the rationale for data collection; future use of data; rights to confidentiality and anonymity; rights to withdraw from the study; protections against adverse consequences in terms of future health care utilization; and review of other potential risks and benefits of study participation. Consenting individuals signed or provided an inked thumbprint on a consent form. Participants then completed an exit interview at the facility where they received PAC, either in a private room or immediately outside the facility.

Enrollment followed a consecutive sampling approach: every client that met the inclusion criteria was asked to participate until an acceptable sample size was achieved. During data collection, interviewers approached 436 eligible PAC clients to request their participation and 412 enrolled in the study. After the exit interview, a subset of 30 participants provided informed consent for an IDI, which was conducted in the same place as the exit interview.

### Analytical Steps

Client data from the questionnaires were entered into an Epi Info database through “double entry” to ensure accuracy. We transferred data into Stata version 14 for statistical analysis. We first carried out a descriptive analysis by estimating the proportions and means for variables used in our analysis. To compare the satisfaction of women that received PAC at referral hospitals and lower-level facilities, we tested for associations, using Pearson chi-square tests, between client characteristics, characteristics of care received, and client satisfaction with the type of facility where they received PAC.

To identify the main drivers of clients’ satisfaction with PAC, we conducted an additional analysis of the questionnaire data collected at all study sites. We first constructed the dependent variable and conducted Pearson chi-square tests (1-sided significance) to assess differences between proportions of clients grouped according to overall satisfaction score (1–4, very low to very high). We used several independent variables in these analyses: participants’ sociodemographic characteristics, reproductive health backgrounds, care-seeking experiences, and PAC visit features. We then estimated ordinal logistic regression models, using cluster-robust standard error estimates to account for the dependent nature of our data caused by recruiting multiple women at the same facilities. With this model, we assessed the relationship between each independent variable that demonstrated significance earlier and the dependent variable. Bivariate tests were conducted, and those with significance of α≤.05 were subsequently analyzed in multivariate models with the same dependent variable. In multivariate tests, we followed an additive procedure to minimize the number of variables, while maximizing the model’s accuracy. Again, our a priori significance level was α=.05.

To understand client perspectives, we analyzed the IDI. First, we used Frameworks Analysis^21^ to identify analytical categories about clients’ perceptions of quality. Then, the team developed a codebook, assigning codes to each framework and conducted a “grounded theory” analysis,^22^ “open coding” the 30 transcripts. Interrater reliability assessments were conducted to ensure the reliability of coding, and the level of agreement was 93%. We then conducted “axial coding” to integrate codes and develop hypotheses. Afterward, we rearranged coded segments of data to display these hypotheses and identified viable explanations. Lastly, we returned to the transcripts and validated these explanations of clients’ level of satisfaction with PAC.

### Ethical Considerations

The Tanzanian National Institute of Medical Research and the U.S.-based Western Internal Review Board approved the study protocol.

## RESULTS

### Participant Characteristics

The majority of clients were between 20 and 24 years of age, but the mean age was approximately 27 (Table 1). Among the 412 women in the study, 115 had no other children or live births; overall, the mean parity was 2.3 live births. Slightly more than four-fifths of participants were married, and 72% had completed primary school (n=297). Forty-one percent (n=167) of participants earned an income independently, and Christians and Muslims composed roughly half of the participant sample, respectively.

**TABLE 1.** Study Population by Sociodemographic Variables, Mainland Tanzania and Zanzibar

		**Parity**	**Married/In Union**	**Completed Primary School**	**Income-Generating Occupation**^a^	**Christian**	**Muslim**
**Age**	**No. (%)**	**Mean**	**No. (%)**	**No. (%)**	**No. (%)**	**No. (%)**	**No. (%)**
<20	50 (12)	<1.0	28 (56)	37 (74)	15 (30)	18 (36)	32 (64)
20–24	113 (27)	1.0	85 (75)	86 (76)	38 (34)	49 (43)	63 (57)
25–29	82 (20)	2.0	68 (83)	61 (74)	41 (50)	46 (56)	36 (44)
30–34	70 (17)	3.0	62 (89)	51 (73)	34 (33)	34 (49)	36 (51)
35–39	54 (13)	4.3	49 (91)	31 (57)	23 (43)	29 (54)	25 (46)
40–46	30 (7)	5.4	27 (90)	21 (70)	13 (43)	22 (73)	9 (27)
Missing	13 (3)	3.6	13 (100)	10 (77)	3 (23)	10 (77)	3 (23)
Overall	412 (100)	2.3	332 (81)	297 (72)	167 (41)	208 (50)	204 (50)

a Participant had her own job that generated an income. Other participants reported that they were not working or were subsistence farmers or homemakers.

Apart from religion, participants from the mainland and from Zanzibar were fairly similar with regard to mean and median ages, mean parity, being married or in union, completion of primary school, and having income-generating occupations. With regard to participants’ religion, 75% and 14% of mainland participants were Christian and Muslim, respectively, whereas in Zanzibar 6% and 93% were Christian and Muslim, respectively.

Apart from religion, participants from the mainland and from Zanzibar were fairly similar.

The majority of participants waited between 1 and 4 days before seeking care for abortion complications (Table 2). One-quarter of participants waited 5 days or longer. Two-thirds of the women enrolled had been in the first trimester of pregnancy when postabortion complications arose, and 12.4% of participants (n=52) reported an induced abortion. Nearly 1 in 5 of participants (n=78) had had at least 1 previous abortion. Two out of 5 participants (n=165) reported that their pregnancy had been unintended, and 54 of these women had been using a contraceptive method when they conceived. With regard to facility type, 61.4% (n=254) women were admitted to a regional referral facility and 38.6% to a district hospital or health center. Less than one-fifth of participants (n=72; 52 from mainland Tanzania and 20 from Zanzibar) were discharged from PAC having voluntarily chosen a modern contraceptive method.

**TABLE 2.** Study Population by Abortion Characteristic and PAC Visit Variables, Mainland Tanzania and Zanzibar (N=412)

**Measure**	**No. (%)**
**Timing of care seeking after observing danger signs of complication**	
1 day	110 (26.7)
2 days	98 (23.7)
3–4 days	99 (24.0)
≥5 days	105 (25.0)
**Gestational age at onset of complications**	
≤12 weeks	283 (68.7)
13–18 weeks	65 (15.8)
≥19 weeks	64 (15.5)
**Client- Report of Spontaneous or Induced Abortion**	
Induced	52 (12.4)
Spontaneous	360 (87.6)
**Participants’ prior experience**	
Ever had an abortion in the past	78 (18.9)
Ever received PAC for a previous abortion	51 (12.4)
First abortion	334 (81.9)
**Participants’ recall of desire for the pregnancy leading to their need for PAC**	
Pregnancy was intended	236 (57.3)
Pregnancy was unintended	165 (40.1)
Pregnancy was mistimed (desire to delay)	123 (29.9)
Pregnancy was unwanted (desire to limit)	28 (6.8)
**Postabortion fertility preferences**	
Would like to get pregnant immediately	92 (22.3)
Would like to get pregnant within 2 years	120 (29.1)
Would like to get pregnant again after 2 years	100 (24.3)
Would like to cease childbearing completely	43 (10.4)
No clear intention for future childbearing	32 (7.8)
Ever used a modern family planning method	198 (48.1)
**Facility type where PAC was received**	
Regional referral hospital	254 (61.2)
District hospital	138 (33.4)
Health center	20 (4.8)
**Waiting time at facility before receiving care for abortion complication**	
Immediately	140 (34.7)
≤30 minutes	87 (21.6)
31–120 minutes	88 (21.8)
>2 hours	88 (21.8)
**PAC treatment method used**	
MVA	265 (64.3)
Sharp curettage	77 (18.7)
Misoprostol	35 (8.5)
**Received pain medication**	
Yes	215 (52.2)
No	197 (47.8)
**Recall of counseling on emergency treatment procedure**	
MVA clients	106 (40.0)
Sharp curettage clients	30 (39.0)
Misoprostol clients	14 (40.0)
Total	150 (36.4)
**Recall of other counseling messages**	
Post-procedure recovery and self-care practices	57 (13.8)
Post-procedure danger signs	158 (38.4)
STI/HIV	11 (2.7)
Postabortion return to fertility	113 (27.4)
**Recall of postabortion family planning counseling and services**	
Discussed future fertility intentions with provider	55 (13.4)
Discussed contraceptive methods with provider	124 (30.1)
Chose and received a modern postabortion contraceptive method	72 (17.4)
Condom (male)	4 (<1%)
Condom (female)	1 (<1%)
Oral contraceptives	23 (5.6)
Injectable (Depo Provera)	19 (4.6)
Implant	5 (1.2)
Intrauterine device	18 (4.3)
Tubal ligation	2 (<1)
Chose a traditional method	6 (1.5)

Abbreviations: MVA, manual vacuum aspiration; PAC, postabortion care; STI, sexually transmitted infection.

### Services and Client Satisfaction

Table 3 presents PAC clients’ ranking of their satisfaction with specific aspects of PAC, with clients’ classification serving as the dependent variable. By combining participants’ scores for the individual measures of satisfaction, we constructed a summative scale to reflect clients’ overall satisfaction with PAC. Each point (1–4) represented an ordered response category defined by participants’ relative evaluation of PAC services. The mean composite score on the summative scale was 2.6, and the median and mode were 2.75. Among all participants, 28% (n=115) and 20% (n=83) were classified into the first and second categories of our ordered response variable, respectively, and 32% (n=131) and 20% (n=83) of clients in the third and fourth.

**TABLE 3.** Study Population by Likert Scale Measures on Client Satisfaction, Mainland Tanzania and Zanzibar (N=412)

		**No. (%)**			
**Satisfaction Measure**		**Very Low (1)**	**Low (2)**	**High (3)**	**Very High (4)**
1	Waiting time before receiving care	42 (10)	12 (3)	63 (16)	292 (71)
2	Privacy of counseling and procedure	31 (8)	9 (2)	55 (14)	312 (77)
3	Cleanliness of facility/PAC room	21 (5)	19 (5)	111 (27)	258 (63)
4	Organization of services	15 (4)	12 (3)	78 (19)	304 (74)
5	Treatment from staff of the facility	9 (2)	12 (3)	58 (14)	327 (81)
6	Treatment from the PAC provider	17 (4)	2 (<1)	45 (11)	344 (84)
7	Clarity, thoroughness of counseling	46 (11)	17 (4)	67 (16)	279 (68)
8	Contraceptive counseling and access to methods	66 (16)	23 (6)	60 (15)	260 (64)
9	Perception of confidentiality of client information.	35 (9)	21 (5)	47 (11)	306 (75)
10	Technical skills of PAC provider	49 (12)	10 (2)	111 (27)	240 (58)
11	Agreement to recommend service to a friend	33 (8)	—	18 (4)	356 (87)
12	Agreement to come back to the same facility for the service again	20 (5)	1 (>1)	16 (4)	340 (90)
Composite score on satisfaction^a^		115 (28)^b^	83 (20)^c^	131 (32)^d^	83 (20)^e^

Abbreviation: PAC, postabortion care.

a Cronbach’s alpha coefficient was calculated to validate the scale (α=.80).

b Mean score of 1–12, ≤2.5.

c Mean score of 1–12, >2.5 and ≤2.67.

d Mean score of 1–12, >2.67 and ≤2.75.

e Mean score of 1–12, >2.75.

Characteristics of the PAC received at referral and lower-level sites are presented in Table 4. Almost one-third of participants admitted at regional hospitals waited for over 2 hours before receiving PAC, whereas only 7.6% of those admitted at lower-level sites waited that long (*P*<.001). The use of PAC treatment methods varied significantly by facility type (*P*<.001), but no difference was found in appropriate use. Participants were more likely to receive pain relief medication at referral hospitals than at lower-level facilities, where 43.7% reported receiving such treatment (*P*<.05). Participants’ recall of postabortion counseling was poor at both types of facilities and varied significantly for the following: counseling on the PAC treatment method (*P*<.05), post-procedure complications and danger signs (*P*<.001), and participants’ return to fertility after abortion (*P*<.05). Concerning postabortion family planning counseling, clients at referral and lower-level facilities varied significantly in terms of discussing fertility desires (*P*<.001), contraceptive methods (*P*<.001), and voluntary method uptake (*P*<.001). Overall client satisfaction with PAC differed significantly based on facility type (*P*<.001).

**TABLE 4.** Service Characteristics and Client Satisfaction at High- and Lower-Level Facilities, Mainland Tanzania and Zanzibar

	**Regional Hospital (N=254)**	**District Hospital or Health Center (N=158)**
**Service Characteristics and Client Satisfaction**	**No. (%)**	**No. (%)**
Waiting time at facility before receiving care**		
Immediately	82 (33.3)	58 (36.9)
≤30 minutes	43 (17.5)	45 (28.7)
30–120 minutes	45 (18.3)	42 (26.8)
>120 minutes	76 (30.9)	12 (7.6)
PAC treatment method used**		
MVA	169 (72.8)	96 (65.8)
Sharp curettage	53 (22.8)	25 (17.1)
Misoprostol	10 (4.3)	25 (17.1)
Received pain relief medication*		
Yes	146 (57.4)	69 (43.7)
No	108 (42.6)	89 (56.3)
Recalled receiving counseling on treatment procedure*		
Yes	88 (34.6)	70 (44.3)
No	166 (65.4)	88 (55.7)
Recalled receiving counseling on post-procedure danger signs**		
Yes	114 (44.9)	27.9 (44)
No	140 (55.1)	114 (72.1)
**Recalled receiving counseling on HIV/STIs**		
Yes	8 (3.2)	3 (1.9)
No	246 (96.8)	155 (98.1)
Recalled receiving counseling on postabortion fertility*		
Yes	60 (23.6)	53 (33.5)
No	194 (76.4)	105 (66.5)
Recalled discussing fertility intentions**		
Yes	16 (6.3)	39 (24.7)
No	238 (93.7)	119 (75.3)
Recalled receiving counseling on contraceptive methods**		
Yes	50 (19.7)	68 (43.0)
No	204 (80.3)	90 (57.0)
Chose and received a modern family planning method**		
Yes	24 (9.5)	48 (30.4)
No	230 (90.5)	110 (69.6)
Composite satisfaction score**		
Very low	92 (36.2)	26 (16.4)
Low	48 (18.9)	35 (22.2)
High	76 (29.9)	55 (34.8)
Very high	38 (15.0)	42 (26.6)

Abbreviations: MVA, manual vacuum aspiration; STI, sexually transmitted infection.

* *P*<.05;

** *P*<.001.

Overall client satisfaction with PAC differed significantly based on facility type.

### Factors Associated With Client Satisfaction With PAC

Table 5 shows the variables included in the multivariate ordinal logistics regression analysis based on their significance in bivariate tests. Participants with at least 1 earlier live birth were more likely to be satisfied with the services they received (odds ratio [OR]=1.66; *P*=.02). Those that received PAC at a district hospital or health center were more likely to be satisfied with PAC than participants who received care at a regional hospital (OR=1.79; *P*=.001). The amount of time clients waited to receive PAC after admission significantly influenced their evaluation of the service, with those who waited for 30 minutes or longer being less likely to be satisfied (OR=0.74; *P*=.02). Relative to participants treated with MVA, those PAC clients who received sharp curettage or misoprostol for PAC were less likely to be satisfied (OR=0.69; *P*=.01). Clients’ recollection of counseling information, receipt of voluntary contraceptive services, and pain relief medication did not significantly influence their satisfaction with PAC.

**TABLE 5.** Factors Associated With Experience of Service Quality: Multivariate Analysis

**Measure**	**OR (95% CI)**	***P* Value**
**Parity**		
No children	Reference	
≥1 child	1.66 (1.10, 2.52)	.02
**Time to facility**		
≤30 minutes	Reference	
>30 minutes	1.09 (0.69, 1.53)	.72
**Facility type**		
Referral hospital	Reference	
Lower-level site	1.79 (1.27, 2.56)	.001
**Time waited at the facility prior to seeing a provider**		
≤30 minutes	Reference	
>30 minutes	0.74 (0.58, 0.93)	.02
**PAC treatment method**		
MVA	Reference	
Other evacuation method	0.69 (0.51, 0.92)	.01
**Received pain relief medication**		
Yes	Reference	
No	0.90 (0.61, 1.32)	.58
**Recalled counseling information on PAC treatment procedure**		
No	Reference	
Yes	1.01 (0.67, 1.50)	.98
**Recalled counseling information on contraceptive methods**		
No	Reference	
Yes	1.05 (0.67, 1.65)	.81
**Chose and received a modern family planning method**		
No	Reference	
Yes	1.23 (0.98, 1.92)	.28

Abbreviations: CI, confidence interval; MVA, manual vacuum aspiration; OR, odds ratio.

### Client Perceptions of Quality

IDI participants (n=30) offered diverse views of satisfaction with PAC and perceptions of the quality of care. The most ardent and prevalent suggestions for service improvements, at times expressed as lamentations, concerned pain experienced during treatment.

I experienced a very painful moment. It was very painful while he (the doctor) had told me there couldn’t be such a severe pain. (MVA for PAC at 10 weeks gestation at district hospital, Mwanza)

IDI participants offered diverse views of PAC satisfaction and perceptions of its quality.

Clients frequently reported that they had to pay for pain relief medication and sometimes leave the PAC treatment area to purchase it from the pharmacy and come back. As illustrated by the preceding quotation, women reported receiving inaccurate information and counseling on the evacuation procedure, not only concerning pain, but in general, prior to receiving treatment.

Clients frequently complained about the lack of thoroughness of post-procedure information provision and counseling. Counseling was often condensed into the brief period in the treatment room immediately after a procedure. For example, when trying to recall the post-treatment conversation she had with her provider, one participant responded.

I can’t tell you. I had an injection and I was floating. (MVA for PAC at 8 weeks gestation, regional referral hospital, Zanzibar)

Clients frequently felt uncomfortable raising questions or expressing themselves when interacting with facility staff, especially their PAC providers.

One thing which I wished to see was my child although it was already dead. I wanted to see how it was and when I wanted to tell the doctor, I thought that I was going to disappoint him so I had to keep quiet. (MVA for PAC, district hospital, Zanzibar)

Aspects of counseling that IDI participants felt were absent included information about postabortion fertility and contraceptive options. Clients ubiquitously reported confusion about when they could become pregnant again. Clients understood the imperative to delay future childbearing for 6 months, but not that fertility could resume as early as 10–14 days after the procedure.

[The providers] told me after 6 months if I have not used any family planning, and conceive I will get miscarriage, so probably in the coming 6 months I better use family planning otherwise if I conceive it will abort again. I have understood them, inshallah, but I have never used family planning. (Misoprostol for PAC, regional referral hospital, Zanzibar)

Counseling that IDI participants felt was lacking included information about postabortion fertility and contraceptive options.

Clients also expressed a need for information about how contraception works, for example, “to know if you are supposed to be investigated before getting the injection … to know how to take those pills” (MVA for PAC, district hospital, Mwanza). Provider attitudes concerning clients’ prerogative to regulate their fertility affected postabortion family planning services:

I wished [the PAC provider] advised me between injection and pills which is the best…but they told me if your husband was here we could give you one right here, but because he is not around, they told me to wait, that I should not start taking family planning drugs now. (MVA for PAC, district hospital, Mwanza)

Some IDI respondents expressed dissatisfaction about excessive wait time for PAC in the facility. One respondent arrived at reception and “wasted much time there” because, “There were too many people, there was a jam” (MVA for PAC, regional referral hospital, Zanzibar). Others reported having to wait for care due to lack of funds:

They asked me if I had money. I told them I don’t have money now, until my relatives come. So he said go and let’s see until they come. I stayed and waited and they did not come. They asked me if they are not coming and told me to then come for cleaning. (MVA for PAC, district hospital, Mwanza).

Clients’ critical commentary was tempered by praise for their providers, whom they regarded with warmth and gratitude. One client felt “[the provider] was good because he was wise and encouraging to me, I felt so good” (MVA for PAC, health center, Mwanza). Clients were relieved and grateful to receive lifesaving PAC treatment, and dissatisfaction with aspects of the care did not affect their appraisal of staff. As one said, “I came here with a problem and it was removed. … so what was supposed to be done has been done!” (MVA for PAC, health center, Zanzibar). At times, clients juxtaposed their tribute to their providers with reports of physical and emotional discomfort. One reported:

I am grateful to him and my God. … I am thankful … they gave a good service. … I had severe pain, however, I endured … tolerated. … I did not tell him anything. … I was just looking at him. (Misoprostol for PAC at 10 weeks gestation, regional referral hospital, Zanzibar)

## DISCUSSION

In this study, we sought to understand how women experience PAC provided by the government of Tanzania, how they view the quality of care, and the factors that contribute to their overall evaluation of the service. We found mixed results. Although high percentages of women were satisfied with the privacy and organization of care and felt they were treated well, participants also identified important areas for improvement. These areas included the cleanliness of the PAC setting, the thoroughness and clarity of counseling, access to voluntary contraception, and the perceived technical skills of the provider. A comparison of referral facilities with lower-level facilities identified the need to address long wait times, particularly at referral facilities, and an overuse of sharp curettage for PAC treatment. The comparison also highlighted irregular use of pain medication and women’s poor recall of critical counseling information, such as on the PAC treatment procedure, postabortion fertility, and contraception options. Our multivariate analysis indicated that parous women tended to view the quality of PAC more favorably than nulliparous women. Similarly, quality was rated more highly by women who received PAC at lower-level sites versus referral facilities, received MVA rather than sharp curettage or misoprostol for PAC, and received PAC within 30 minutes of admission.

High percentages of women were satisfied with the PAC received and felt they were treated well, but they also identified areas for improvement.

Our qualitative results contextualized these findings. Women’s almost unanimous appreciation and praise for the health care workers providing the service was interwoven with expressions of hardship. These expressions highlighted experiences of pain during PAC treatment, difficulties concerning demands for service fees, dissatisfaction and poor recall of desired counseling information, disappointment with the contraception information and method options available, and long wait times.

The government PAC in mainland Tanzania and in Zanzibar should address the gaps in delivery of pain medication. According to policies throughout Tanzania, public-sector facilities cannot charge user fees for PAC. While our study facilities appeared to follow this directive, many participants had to pay for pain relief medication as a separate service component. This factor may not have emerged from a study that only used quantitative data. Studies on health care worker behavior and performance in the context of maternal health care in Tanzania have reported that the government has poor responsiveness to the workers’ financial reimbursement claims for needed items that are not available through the public-sector supply chain. The workers may in turn resort to officially unpermitted user fees to maintain financial resources at the facility.^23^ These studies and others in different countries describe how excessive workload, lack of time, lack of training on the need for pain medication, and insufficient knowledge about pain management among nurses and midwives might also explain these gaps in PAC provision.^24^^,^^25^ Training and practice, particularly at lower-level facilities (e.g., district hospitals), should emphasize pain management as a fundamental component of PAC. Review and possibly revision of curricula, training, and quality assurance materials should direct such guidance to those that perform PAC and those working at the pharmacy and inventory levels that regulate the availability of medical supplies. Broader efforts are also needed to address the structural constraints to improving the public-sector supply chain and the financial resources at the site level to prevent health care workers from imposing inappropriate user fees.

We found that counseling on PAC treatment procedures and postabortion fertility, typical danger signs, and contraceptive eligibility, particularly in referral facilities, was generally poor. Similar to receipt of pain relief medication, these factors did not appear as significant drivers of satisfaction in statistical analysis; however, comparisons of performance by facility type suggested that efforts must be made at multiple levels to ensure that counseling addresses clients’ informational and interpersonal needs. Weaknesses in counseling during PAC appear to hinge on lack of training of providers in communication and dealing with clients’ feelings, insufficient time for counseling, lack of integration of counseling into other routine client-provider interactions, and provider biases against discussing abortion.^26^

Counseling on PAC treatment procedures and postabortion fertility, typical danger signs, and contraceptive eligibility was generally poor.

Without adequate counseling on procedures and options for PAC treatment, practitioners in our study overused sharp curettage for PAC clients in their first trimester. We also found that the type of PAC treatment received influenced women’s satisfaction with PAC. Women with incomplete abortion who did not receive MVA usually had sharp curettage, which is relatively invasive and painful; therefore, these women tended to have lower levels of satisfaction than women treated with MVA. Zanzibar, where the government has a policy approving misoprostol for PAC, has not adopted clinical guidelines on using it to treat abortion complications. Consequently, providers’ knowledge on its use for PAC is adapted from protocols for other conditions, such as postpartum hemorrhage. Considering the demand for PAC and availability of both surgical and medical treatment, women in Zanzibar should be given the choice between MVA and misoprostol for PAC treatment. Further, providers should be oriented to relevant guidelines on misoprostol use for PAC, including accurate dosing according to specific indications and route of administration.

Among the various sociodemographic factors examined, only parity emerged as being significant in our multivariate analysis of client satisfaction. Several factors could explain this result, including PAC not being youth friendly. Survey data on and qualitative narratives offered by multigravida clients depicted these women as being relatively older, married, and more experienced with the health system and obstetrical care, and having firmer preferences about fertility intentions and contraceptive use. This corroborates with others from studies on women’s satisfaction with maternal health care. One study, in Sri Lanka, reported that multiparous women were more likely than primiparous counterparts to evaluate perinatal services highly, likely owing to their relative experience and realistic expectations.^27^ A qualitative study on decision making and experiences seeking maternal care in Sierra Leone had similar findings, remarking that multigravida women appeared to have more autonomy and perceived control during their interactions with the health care system.^28^ Altogether, these findings suggest that efforts concerning PAC should emphasize meeting the needs of younger women with relatively little childbearing experience.

A large majority of clients in this study were enrolled at regional referral hospitals, indicating a need to revisit and potentially revise the processes used to decentralize PAC in the study areas. These facilities performed relatively poorly in terms of counseling on PAC treatment and family planning, postabortion contraceptive provision, clients’ waiting times, and clients’ overall satisfaction. Other studies have shown that clients often bypass primary care facilities for regional hospitals in Tanzania (often at great expense), providers in rural areas tend to be less skilled than those in urban areas, and patients are aware of these deficiencies.^29^^,^^30^ Our findings suggest that redoubling efforts to decentralize services should also include improving quality at the tertiary level. In fact, the most robust predictor of clients’ satisfaction was where they received PAC. Women treated for an incomplete abortion at a regional referral hospital were significantly less likely to be satisfied with PAC than those at lower-level sites. Previous research conducted at the same facilities highlighted factors that might explain this finding, including the facilities’ congestion, longer waiting periods, greater pressure on providers to multi-task and deal with higher case burdens, and the isolation of family planning services to separate sections of facilities.^31^ Future research and training programs should emphasize technical quality, communication, and service integration at all levels of care as service coverage expands from tertiary to lower-level facilities.^32^ Additional research should document the systems requirements for sustaining quality as coverage expands, first at higher-level facilities, and establish frameworks for scaling up to intermediate and primary sites accordingly.^33^

A need exists to revisit and potentially revise the processes used to decentralize PAC in the study areas.

Our analysis also illustrates the complexity of understanding quality of care from clients’ perspective. Clients generally offer moderate to high appraisals of service quality when scoring their perceptions of quality quantitatively, despite their qualitative feedback largely focusing on dissatisfactory aspects of their experience with PAC. Although we cannot attribute these discrepancies to any particular factor in our study environment, previous research has illustrated how patients often positively reinterpret negative aspects of health care for various reasons including fear of reprisal, a sense of dependency on medical professionals, and expectations of etiquette.^34^ Such findings underscore the importance of qualitative research. In this study, it helped us identify differences between clients’ ratings of health care quality, their desires, and how they felt during care. The health system should respond by reviewing standards of care and improving implementation to address client needs. Based on PAC research and program experience, providing written guidance to PAC clients as well as information on postabortion family planning, self-care, and when to seek health care improves the client experience and compels providers to comply with standards of care.^35^ Program evaluators should use qualitative data and adapt quantitative measures for service quality that reflect clients’ actual preferences and desires.^36^

Of particular note, this study revealed extremely low levels of contraceptive provision and voluntary uptake among women at the study sites in both mainland Tanzania and in Zanzibar. Only 17% of the women actually received a modern contraceptive method and fewer than 7% received a voluntary LARC method. Quality gaps of this magnitude likely involve multiple factors, such as informal task-sharing arrangements, staff being unfamiliar or unqualified to implement protocols on family planning counseling and provision of a variety of methods, and providers’ time constraints and biases. Such biases may particularly exist toward young and nulliparous clients, who made up 28% of our study sample. This finding corroborates a larger analysis based on 4 years of data from 10 countries in sub-Saharan Africa that found contraceptive uptake was less likely among women younger than 20 years old compared with those over 25 years.^37^ Additional reasons could include weak logistics systems that fail to consider the demand for contraceptives outside typical family planning settings and the tendency of quality assurance schemes to overlook PAC, including postabortion family planning, as services requiring routine supervision and follow-up. These problems being most observed in referral-level facilities mirrors findings from another meta-study that identified similar associations between care at primary-care facilities and contraceptive uptake in several countries.^38^

This study revealed extremely low levels of contraceptive provision and voluntary uptake at the study sites.

### Limitations

The study has some limitations. It compared women’s experiences with PAC at different levels of the health system, but it only enrolled 20 women from health centers. To achieve sampling targets within timelines, it was necessary to enroll participants at sites where most women were seeking care rather than wait extensively to enroll participants at low-volume sites. Similarly, the study was unable to obtain a sample that contained robust strata by evacuation method type, most PAC cases involved surgical performance of MVA, with markedly fewer using curettage and medical techniques. Consequently, closer examination of women’s preferences in terms of PAC treatment method was not possible. Because this study was intended to provide formative knowledge for a program to be implemented in specific regions of the country, it did not employ national probability sampling; therefore, findings cannot be generalized to the entire country. In Tanzania, PAC is also available from the private sector, and we only assessed it in public facilities. Thus, our results are not generalizable to women seeking PAC in the private sector. Our findings provide information for quality improvement interventions in settings identified by the government of Tanzania. In all settings, it is important to note the potential influence of courtesy bias on participants’ responses. They might not have felt at liberty or comfortable rendering negative feedback about the care they received, a limitation common in research on this topic.^39^ Financial considerations limited the volume of data that we could collect, making it necessary to pool data from mainland and Zanzibar for our analysis. This approach may conceal important regional differences about PAC provision.

## CONCLUSIONS

Overall, the study reveals considerable room for improvement in the public-health sector PAC program in our mainland sites in Mwanza and Geita regions and those in Zanzibar. We identified noteworthy discrepancies in the client-reported quality of PAC at referral-level and lower-level facilities. Although clear areas for improvement existed at all sites, women were less likely to report satisfaction with care at referral facilities owing primarily to inadequate counseling, delays in receiving PAC treatment after admission, and poor emphasis on postabortion fertility, family planning information, and contraceptive provision. Strategies to scale up PAC must encompass solutions to these problems and the capacity of the local health systems to sustain quality of care wherever PAC is introduced. Nevertheless, women’s rating of overall service quality demonstrates moderate levels of satisfaction. Relevant factors were the women’s parity, the type of facility where they received PAC, delay in receiving care, and the PAC treatment method used. The clients’ narratives about their experiences receiving PAC provides a richer picture of what mattered to them. When asked to score their satisfaction, women expressed decisions that were often inconsistent with deeply felt desires and emotional reactions to using PAC. Understanding clients’ preferences, expectations, and wishes is vital toward strengthening health care to address underutilization of services in general, including PAC. Improving the areas that fell short in this study will translate to critical progress for women’s health in these regions.

Strategies to scale up PAC must encompass solutions to problems and the capacity of the local health systems to sustain quality of care.

In terms of policy and programming, our findings provide relevant implications. In particular, training, whether centralized or on the job, and other supervision and quality assurance systems should emphasize strengthening capacity to ensure and monitor availability of a wide range of contraceptive methods in PAC settings, as well as the quality of family planning counseling, especially at tertiary facilities. In addition, the need for access to PAC treatment procedures merits attention in both the mainland and in Zanzibar. Where misoprostol is permitted for PAC, policy makers and technical assistance partners should develop guidelines and strengthen capacity among providers to deliver medical methods appropriately. They should also ensure postabortion family planning is offered equally to PAC clients regardless of the method of treatment they receive. Where possible, supporting task sharing to mid-level providers and decentralizing care to lower level health facilities can make PAC more available to women closer to where they live. In all cases, protocols, training and supervision tools, and practices need to emphasize pain management as an essential component of PAC. Ongoing efforts by the government and technical assistance partners should adopt a focus on the preferences, needs, and perceived barriers to PAC including family planning among youth and emphasize approaches that address those factors in their partnership with health care workers and managers at PAC service delivery points across the country.

Policy and programmatic implications notwithstanding, continued mixed-method research is necessary to explore how service delivery and client preferences shape satisfaction and decision making on use of PAC. Programs that integrate such work into routine monitoring and evaluation activities position themselves for improvements that are guided by clients’ expressed perspectives and needs.

## Notes

**Peer Reviewed**

**Funding:** This manuscript was generated from a study conducted under the Post Abortion Care Project in Tanzania funded by the United States Agency for International Development under agreement AID-OAA-A-00050.
